# Pass the salt: physiological consequences of ecologically relevant hyposmotic exposure in juvenile gummy sharks (*Mustelus antarcticus*) and school sharks (*Galeorhinus galeus*)

**DOI:** 10.1093/conphys/cow036

**Published:** 2016-10-06

**Authors:** Andrea J. Morash, Sara R. C. Mackellar, Louise Tunnah, David A. Barnett, Kilian M. Stehfest, Jayson M. Semmens, Suzanne Currie

**Affiliations:** 1 Department of Biology, Mount Allison University, Sackville, New Brunswick, Canada E4L 1G7; 2 Atlantic Cancer Research Institute, Moncton, New Brunswick, CanadaE1C 8X3; 3 Fisheries and Aquaculture Center, Institute for Marine and Antarctic Studies, University of Tasmania, Hobart, Tasmania, 7053Australia

**Keywords:** Climate change, conservation, estuarine, hyposalinity, sharks

## Abstract

Estuarine habitats are frequently used as nurseries by elasmobranch species for their protection and abundant resources; however, global climate change is increasing the frequency and severity of environmental challenges in these estuaries that may negatively affect elasmobranch physiology. Hyposmotic events are particularly challenging for marine sharks that osmoconform, and species-specific tolerances are not well known. Therefore, we sought to determine the effects of an acute (48 h) ecologically relevant hyposmotic event (25.8 ppt) on the physiology of two juvenile shark species, namely the school shark (*Galeorhinus galeus*), listed by the Australian Environmental Protection and Biodiversity Conservation Act as ‘conservation dependent’, and the gummy shark (*Mustelus antarcticus*), from the Pittwater Estuary (Australia). In both species, we observed a decrease in plasma osmolality brought about by selective losses of NaCl, urea and trimethylamine *N*-oxide, as well as decreases in haemoglobin, haematocrit and routine oxygen consumption. Heat-shock protein levels varied between species during the exposure, but we found no evidence of protein damage in any of the tissues tested. Although both species seemed to be able to cope with this level of osmotic challenge, overall the school sharks exhibited higher gill Na^+^/K^+^-ATPase activity and ubiquitin concentrations in routine and experimental conditions, a larger heat-shock protein response and a smaller decrease in routine oxygen consumption during the hyposmotic exposure, suggesting that there are species-specific responses that could potentially affect their ability to withstand longer or more severe changes in salinity. Emerging evidence from acoustic monitoring of sharks has indicated variability in the species found in the Pittwater Estuary during hyposmotic events, and together, our data may help to predict species abundance and distribution in the face of future global climate change.

## Abstract (precis)

Estuaries are critical nursery grounds for juvenile sharks; however these shallow waters are susceptible to extreme fluctuations in salinity. We investigated the physiological effects of an ecologically relevant low saline exposure on two inshore juvenile shark species. School sharks had a more dramatic response, possibly influencing their distribution and abundance.

## Introduction

Shallow estuarine habitats provide safe nursery conditions for many elasmobranchs by offering protection from larger predator species and abundant resources for growth and development (Branstetter, 1990; Castro, 1993; Heupel *et al*., 2007). However, these estuarine environments are subject to large fluctuations in abiotic factors, leaving these animals vulnerable to a variety of stressors. Rainfall events, for example, can potentially decrease salinity, creating a hyposmotic environment. Average rainfall events are not likely to elicit large decreases in salinity; however, heavy rainfall associated with extreme weather events can severely dilute estuaries over short periods of time (i.e. hours), and rapid and dramatic changes in the local environment directly impact elasmobranch behaviour (Heupel *et al*., 2003; Udyawer *et al*., 2013). Recent climate change predictions suggest that the number of extreme weather events is on the rise (IPCC, 2013), potentially increasing the frequency and severity of hyposmotic stress events for juvenile sharks residing in estuaries.

Changes in salinity are problematic for most marine shark species because they are osmoconformers (Smith, 1931), but species-specific tolerances to salinity change are not well studied. Elasmobranchs typically use urea and trimethylamine *N*-oxide (TMAO) to regulate osmolality, and it is well documented that a loss of one or both osmolytes occurs during exposure to hyposmotic environments (Schmidt-Nielsen *et al*., 1972; Forster and Goldstein, 1976; Dowd *et al*., 2010; Guffey and Goss, 2014; MacLellan *et al*., 2015). TMAO is not only an important osmolyte, it also acts as a chemical chaperone and helps to protect against protein damage (Yancey, 2005). Therefore, during a hyposmotic event and a loss of TMAO and subsequent change in the urea-to-TMAO ratio, there is potential for increased protein damage and cellular stress. To counteract this loss in chemical chaperoning, heat shock proteins (HSPs), the highly conserved and ubiquitous molecular chaperones (Georgopoulos and Welch, 1993), help to protect against protein aggregation and misfolding during hyposmotic stress, but at an increased energetic cost (Kumar, 2009). Indeed, in response to a 30% decrease in salinity, spiny dogfish sharks (*Squalus acanthias*) lost TMAO from their gills, showed signs of protein damage in this tissue and significantly induced gill HSP70 protein (MacLellan *et al*., 2015). The ability of some species of sharks to protect against cellular stress from hyposmotic environments could allow them to remain in the estuarine environment and avoid predation in deeper water.

The Pittwater Estuary, part of a designated shark refuge area, located in southeast Tasmania, Australia, exhibits large changes in salinity with freshwater input from the Coal River and seawater influx from the adjoining Frederick Henry Bay. The salinity normally varies between 34 and 37 ppt during the summer months, but during a wet summer the salinity can drop to ~25.5–28 ppt as a result of rainfall (Stevens and West, 1997). To illustrate, in 2013, there were seven hyposmotic events when the salinity was below 28 ppt for 48 h or more (Marine Culture Pty. Ltd; measured at ~30 cm below the surface). Salinity is a significant driver of movement and distribution of inshore sharks and rays (see Schlaff *et al*., 2014 and Yates *et al*., 2015 for review), and population studies in the Pittwater Estuary show that the number of young of the year sharks (0+) declines during wet summers, suggesting that salinities in this range may be physiologically challenging, leading them to move out of the nursery grounds into deeper water (Stevens and West, 1997). The Pittwater Estuary is considered a communal estuary and is populated by a variety of shark species, including the school shark (*Galeorhinus galeus*), listed as ‘conservation-dependent’ by the Australian Environmental Protection and Biodiversity Conservation Act, and the gummy shark (*Mustelus antarcticus*). It is unknown how either species responds to ecologically relevant periods of acute, low salinity. It is possible that any differences in physiological tolerance to low salinity may be involved in driving species-specific use of the estuary.

Given that shark nurseries will experience regular bouts of low salinity, we were interested in determining the physiological response to hyposmotic exposure in juvenile school and gummy sharks following an acute drop in salinity, mimicking a rainfall event. We hypothesized physiological changes in both species in response to an ecologically relevant hyposmotic exposure that may influence potential species-specific movement out of the estuary during hyposmotic events. We predicted that both species would show increased signs of cellular stress, protein damage and an inability to maintain metabolic homeostasis. Understanding the physiological responses to salinity change in these juvenile sharks could allow us to estimate the effects of more frequent heavy rainfall events on the population structure of this estuary and may be important for the recovery of the school shark population.

## Materials and methods

### Animal collection and care

Young of the year (0+) school sharks (*G. galeus*; *n* = 10, 45.8 ± 8.9 cm) and 1+ gummy sharks (*M. antarcticus*; *n* = 8, 52.7 ± 18.4 cm) were captured via long-line in two shallow inshore regions, the Pittwater Estuary and Frederick Henry Bay (42.79°S, 147.54°E) outside Hobart, Tasmania, Australia in March 2014. Sharks were immediately transported in aerated seawater to the Institute for Marine and Antarctic Studies in Hobart. Animals were held in large (2 m × 2 m × 1 m) outdoor seawater tanks (34 ppt, ~17°C) for 7 days prior to experimentation. Upon arrival at the laboratory, sharks were fitted with fin tags to allow for individual identification. Water temperature and nitrates were monitored daily. Fish were fed frozen squid every other day, but were fasted for 24 h prior to experimentation. All experiments were approved by the University of Tasmania Animal Ethics Committee, permit number A13796.

### Experimental protocol

The hyposaline exposure at 25.8 ppt (~75% seawater), mimicked the natural rainfall events that are observed in these estuaries (Marine Culture Pty. Ltd). A ~0.7 ml blood sample was taken at time (*t*) = 0 h, and the salinity of the tank was then immediately dropped from 34.3 ppt (100% seawater) to 25.8 ppt (75% seawater) over 3 h (averaging 2.8 ppt h^−1^ decrease) by dilution with fresh water. These hyposmotic conditions were maintained for 48 h, after which time salinity was returned to 100% seawater and sharks were allowed to recover for 24 h. To account for any influence of repeated sampling, we conducted a control repeated sampling experiment on a separate group of sharks prior to the hyposmotic experiment. In the control experiment, the sharks remained freely swimming at 34 ppt (ambient seawater conditions) for 72 h and blood samples were taken at the matching time intervals for the hyposmotic experiment (see below). We did not observe significant effects of time or sampling; thus, control data are not presented here. These control animals were also used in a companion study (Tunnah *et al*., 2016).

### Blood and tissue sampling

Whole blood samples were collected at *t* = 0 h (control, immediately before initiation of salinity drop; school sharks *n* = 10; gummy sharks *n* = 8), *t* = 3 h (upon completion of the salinity drop to 70% seawater; school sharks *n* = 10; gummy sharks *n* = 8), *t* = 27 h (school sharks *n* = 8; gummy sharks *n* = 7), *t* = 48 h (school sharks *n* = 8; gummy sharks *n* = 7) and *t* = 72 h (recovery, 100% seawater; school sharks *n* = 4; gummy sharks *n* = 2) via caudal puncture in restrained animals, a procedure completed in <2 min. Tissues (gill and white muscle) were collected from the control group (school sharks *n* = 3; gummy sharks *n* = 5) and at *t* = 48 h (school sharks *n* = 4; gummy sharks *n* = 3) and *t* = 72 h (school sharks *n* = 4; gummy sharks *n* = 2) from the experimental group. Sharks were terminally sampled after pithing the brain and transecting the spinal cord. Samples were excised, flash frozen in liquid nitrogen and stored at −80°C for subsequent analysis. As this was a field study on wild fish, one of which is listed as ‘conservation dependent’ by the Australian Environmental Protection and Biodiversity Conservation Act, we have uneven sample sizes and a lower replication compared with a laboratory-based study.

### Haematological analysis

Haematocrit was measured in duplicate with a portable field haematocrit centrifuge (Haematokrit 210 centrifuge; Hettich Zentrifugen, Tuttlingen, Germany). Haemoglobin was measured using a HemoCue^®^ Hb 201^+^ system (Hemocue, Ängelholm, Sweden) and corrected for fish blood according to Clark *et al*. (2008). Whole blood glucose and lactate were measured using a OneTouch Ultra glucometer (LifeScan, Milpitas, CA, USA) and a Lactate Pro^TM^ hand-held lactate meter (Arkray Global Business, Inc., Kyoto, Japan), respectively, both of which have been used previously in elasmobranchs and have been validated in school (Awruch *et al*., 2011) and mako shark whole blood (French *et al*., 2015). The remaining whole blood was then centrifuged at 13 000 rpm (17 949***g***) for 4 min to separate plasma and red blood cells (RBCs). Plasma was carefully removed and transferred to a new cryovial; the buffy coat was discarded, and both the plasma and the remaining RBCs were flash frozen in liquid N_2_ and then stored at −80°C for later analyses.

### Plasma osmolality and ions

The osmolalities (in milliosmoles per kilogram) of school and gummy shark plasma, as well as tank water, were measured using a Wescor Vapro 5520 Vapour Pressure Osmometer (Wescor Inc, Logan, UT, USA). Plasma [Na^+^] and [K^+^] (millimolar) and were measured for both species using the SpectrAA 220 Atomic Absorption Spectrometer (AAS) and its accompanying software, SpectraAA 220, version 3.10 (Varian, Mulgrave, Victoria, Australia), and plasma [Cl^−^] (in millimoles per litre) was measured using a M925S Chloride Analyzer (Nelson Jameson Inc., Marshfield, WI, USA) according to the manufacturer's instructions.

### Protein analyses

Soluble protein was extracted from frozen gill and white muscle tissue of both school sharks and gummy sharks as described by Fowler *et al*. (2009). School and gummy shark RBC protein was extracted according to Tunnah *et al*. (2016). Both RBC and tissue protein concentrations were determined using a BioRad DC Protein Assay Kit. The absorbance of samples and bovine serum albumin (BSA; Sigma Aldrich) standards was read in Greiner clear-bottomed 96-well plates at 750 nm using an M5 SpectraMax plate reader and SoftMax Pro software (Molecular Devices, Sunnyvale, CA, USA).

### Heat-shock proteins

The concentrations of HSP70 and HSP90 (in nanograms per microgram of total protein) were determined using immunoblotting. Samples were prepared according to Tunnah *et al*. (2016) and compared with a commercially available standard (recombinant rat HSP70/72, SPP 758; human native HSP90, SPP770; Enzo Life Sciences) to quantify the specific HSP in each sample. For the primary antibody rabbit anti-HSP70/HSC70 (1:5000 dilution of AS05-083A; Agrisera), we used a horseradish peroxidase-tagged goat anti-rabbit secondary antibody to visualize at 1:10 000 dilution (SAB-300; Enzo Life Sciences). For the HSP90 primary antibody (mouse anti-Hsp90; 1:2500 dilution of SMC-107; StressMarq Biosciences Inc.), we used a horseradish peroxidase-tagged goat anti-mouse secondary antibody at 1:5000 dilution (ab5870; Abcam), Cambridge, UK. Protein bands were visualized in Lumigen ECL Ultra (TMA-6) reagents (Southfield, MI, USA) and imaged using a Molecular Imager VersaDoc™MP 400 System (BioRad, Berkley, CA, USA) and Quantity One 1-D Analysis software. Image Lab^®^ software (BioRad) was used for quantifying the band density in each sample.

### Ubiquitin

As an indirect measure of protein damage, ubiquitin levels were measured in RBCs, gill and white muscle using dot blots. Soluble protein (0.5 μg per sample) was blotted onto a nitrocellulose membrane (BioRad), as well as 0.2 μg of ubiquitin standard for relative quantification (catalogue no. sc-111402; Santa Cruz Biotechnology, Dallas, TX, USA). The mouse primary antibody used (1:2500 dilution in 5% BSA/tris-buffered saline with Tween-20; TBS-T) probed only for polyubiquitinylated proteins and not monoubiquitinylated proteins or free ubiquitin (BML-PW8805-0500; Enzo Life Sciences, Farmingdale, NY, USA). The secondary antibody (1:20 000 dilution in 5% BSA/TBS-T) was a goat anti-mouse IgM (ab97230; Abcam, Cambridge, UK). Blots were visualized as for HSPs, and the ubiquitin content was quantified relative to the standard run on each blot.

### Osmolytes

Plasma, gill and white muscle urea concentration (in millimoles per litre) were analysed on perchloric acid-extracted samples using the method of Rahmatullah and Boyde (1980) in quartz cuvettes using a Spectronic Unicam UV1 (Thermo Fisher Scientific, Waltham, MA, USA). Plasma, gill and white muscle TMAO concentrations were determined with liquid chromatography–mass spectrometry (LC-MS) as described by Tunnah *et al*. (2016).

### Na^+^/K^+^-ATPase activity

The activity of the ion symporter Na^+^/K^+^-ATPase (NKA) was measured as described by McCormick (1993) with modifications described by MacLellan *et al*. (2015). This assay assessed any changes in gill NKA function during hyposmotic stress. The soluble protein concentration was determined for each aliquot of gill homogenate, as above.

### Oxygen consumption

Routine metabolic rate was measured in separate groups of school sharks (~450 g) and gummy sharks (~750 g) in control and hyposmotic environments in a similar manner to Tunnah *et al*. (2016) using a Fibox O_2_ probe (PreSens Fibox). The average of two slopes of oxygen depletion over time was used to calculate routine O_2_ consumption, taking into account the background O_2_ consumption rate (measured as oxygen depletion in the empty respirometer), the volume of the chamber, shark mass, temperature and daily barometric pressures.

### Statistical analyses

All statistical analyses were performed using R Studio (version 3.2.1). An α-critical level of 0.05 was used for every test when determining significance. Data generated from repeated sampling (metabolic rate, RBCs and plasma) were analysed using linear mixed models to determine the effect of time (continuous variable for blood data, categorical variable for tissue data) and species and their interaction on all dependent variables. Data and generated residuals were assessed visually for homogeneity of variance and normality. Tukey's *post hoc* test was used to determine where means differed from one another. For tissue data analysis, a two-way fixed-factor (time and species) analysis of variance was performed. Normality (Shapiro–Wilk test) and homogeneity of variance (Levene's test) were assessed for each parameter and the appropriate transformation (log or sqrt) was applied when necessary. As above, Tukey's *post hoc* tests were used to determine which time points differed from each other. Our sample size for the recovery time period did not allow for statistical analysis; however, we have kept the recovery data available in the figures/tables for reader interpretation.

## Results

### Osmoconformation

Hyposmotic conditions resulted in a significant interaction with species and time for plasma osmolality (*P* < 0.0001), and when we split the data by species there was a significant decrease in plasma osmolality for both species over 27 h (Table 1; school ~26%, *P* < 0.01; gummy ~22%, *P* < 0.01). Plasma osmolality remained significantly lower than control values by the 48 h time point for both gummy and school sharks (Table 1).

**Table 1:** Plasma osmolality (in milliosmoles per kilogram) and ion concentrations (in millimoles per litre) in school sharks (*Galeorhinus galeus*) and gummy sharks (*Mustelus antarcticus*) before (0 h) and after exposure to 75% seawater

	Time					
Species	Dependent variable	0 h	3 h	27 h	48 h	Recovery
School shark	Osmolality*	968 ± 6.63 (10)^a^	864 ± 9.32 (10)^b^	735 ± 15.6 (8)^c^	792 ± 4.75 (9)^d^	935 ± 12.6 (3)
	[Na^+^]	328 ± 8.47 (10)^a^	292 ± 8.36 (10)^b^	261 ± 6.62 (8)^c^	277 ± 8.04 (9)^bc^	293 ± 26.7 (4)
	[Cl^−^]*	246 ± 6.63 (10)^a^	226 ± 1.72 (10)^b^	208 ± 2.09 (7)^c^	217 ± 3.26 (9)^bc^	246 ± 17.5 (3)
	[K^+^]	4.55 ± 0.09 (10)^a^	4.42 ± 0.21 (10)^a^	4.44 ± 0.26 (8)^a^	4.91 ± 0.14 (9)^a^	5.91 ± 0.35 (4)
Gummy shark	Osmolality*	994 ± 3.96 (8)^a^	826 ± 13.3 (8)^b^	785 ± 3.25 (7)^c^	765 ± 5.89 (5)^c^	898 ± 6.84 (2)
	[Na^+^]	345 ± 4.51 (8)^a^	282 ± 6.82 (8)^b^	260 ± 12.9 (7)^c^	263 ± 7.14 (5)^bc^	258 ± 5.21 (2)
	[Cl^−^]*	253 ± 3.57 (8)^a^	210 ± 1.7b (8)^b^	201 ± 3.72 (7)^b^	205 ± 4.41 (5)^b^	244 ± 1.00 (2)
	[K^+^]	4.55 ± 0.18 (8)^a^	4.14 ± 0.14 (8)^a^	4.45 ± 0.50 (7)^a^	4.42 ± 0.46 (5)^a^	4.73 ± 0.40 (2)

Data are presented as mean values ± SEM (sample size). Different superscript letters indicate statistical differences (*P* < 0.05) over time. *Significant interaction between species and time.

Plasma sodium concentration of both species decreased by 23% in hyposmotic conditions (Table 1; 0 vs. 27 h, *P* < 0.0001) and did not change after 48 h (0 vs. 48 h, *P* < 0.0001). Gummy and school sharks had different responses to hyposalinity in plasma chloride (*P* = 0.053); however, overall both species showed significant decreases in [Cl^−^] with low-saline exposure (Table 1; *P* < 0.001). Hyposmotic conditions did not affect plasma potassium in either species (Table 1; *P* = 0.15), with no difference between species (*P* = 0.41).

**Figure 1:**
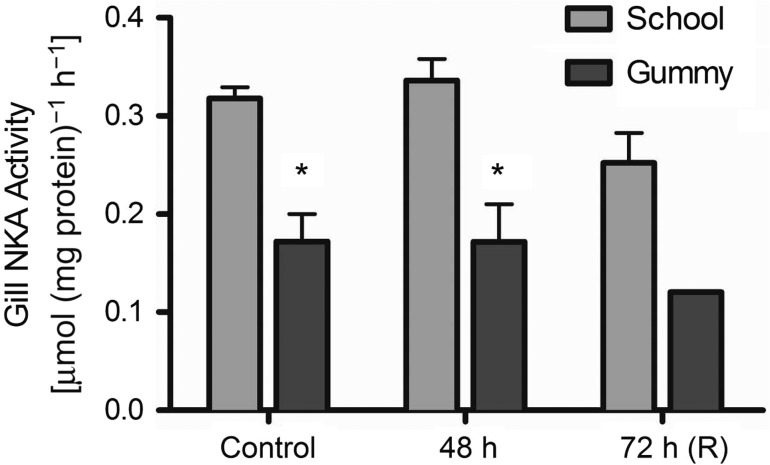
Na^+^/K^+^-ATPase (NKA) activity (in micromoles per milligram of protein per hour; mean values + SEM) in the gill tissue of school sharks (*Galeorhinus galeus*) and gummy sharks (*Mustelus antarcticus*). Asterisks indicate a statistically significant difference between species (*P* < 0.05; see Materials and methods for species replication numbers). Recovery (R) data are not included in the statistical analyses.

We measured the activity of gill NKA activity as a proxy for gill function during hyposmotic exposure. Na^+^/K^+^-ATPase activity was not affected by the 75% seawater treatment in either species (Fig. 1; *P* = 0.178); however, school sharks had consistently higher NKA activity throughout the experiment (*P* < 0.0001).

**Figure 2:**
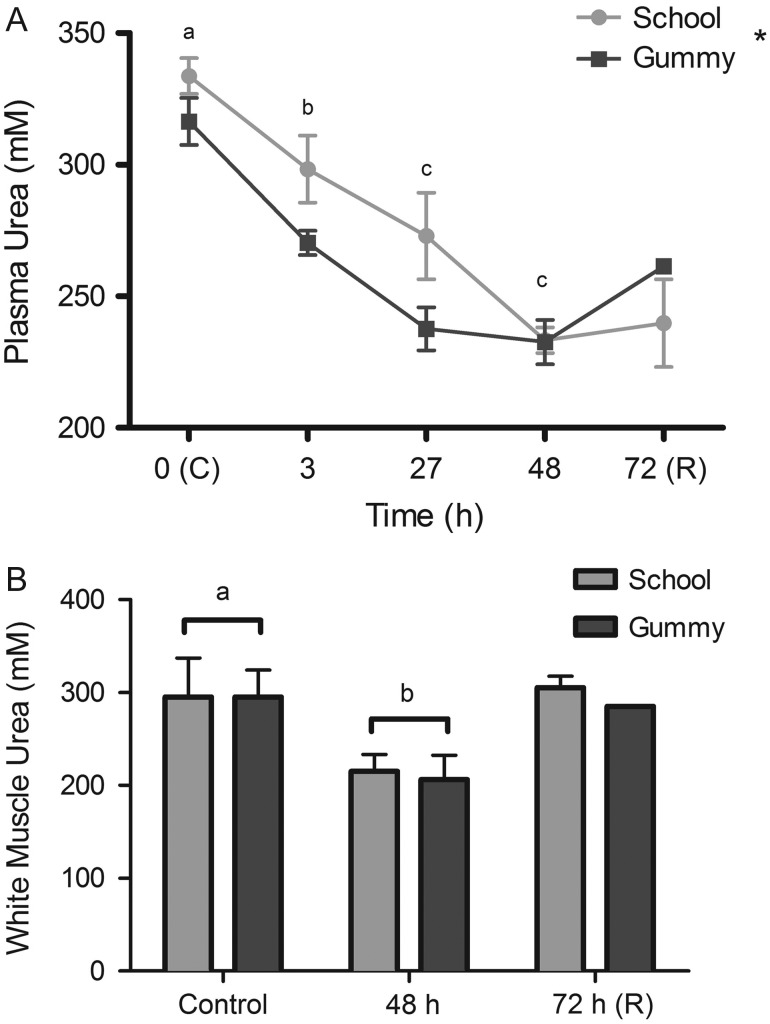
Urea concentration (in millimoles per litre; mean values ± SEM) in the plasma (**A**) and white muscle (**B**) of school sharks (*G. galeus*) and gummy sharks (*M. antarcticus*) following exposure to 75% seawater. Different letters indicate statistically significant differences over time (*P* < 0.05; see Materials and methods for species replication numbers). Asterisks indicate a statistically significant effect of species (*P* < 0.05). Recovery (R) data are not included in the statistical analyses.

The 75% seawater resulted in significant decreases in plasma urea concentration over time (~28% overall decrease, *P* < 0.001; Fig. 2A) in both species. White muscle urea concentration also decreased by 29% in both species after 48 h in 75% seawater (Fig. 2B; control vs. 48 h, *P* = 0.0133). Unlike plasma and white muscle urea, gill urea did not change over time (data not shown; *P* = 0.53) and was not different between species (*P* = 0.07). At the control time point, gill urea concentration in the school shark was 222 ± 35.5 mmol l^−1^, and in the gummy shark it was 303 ± 56.0 mmol l^−1^.

**Figure 3:**
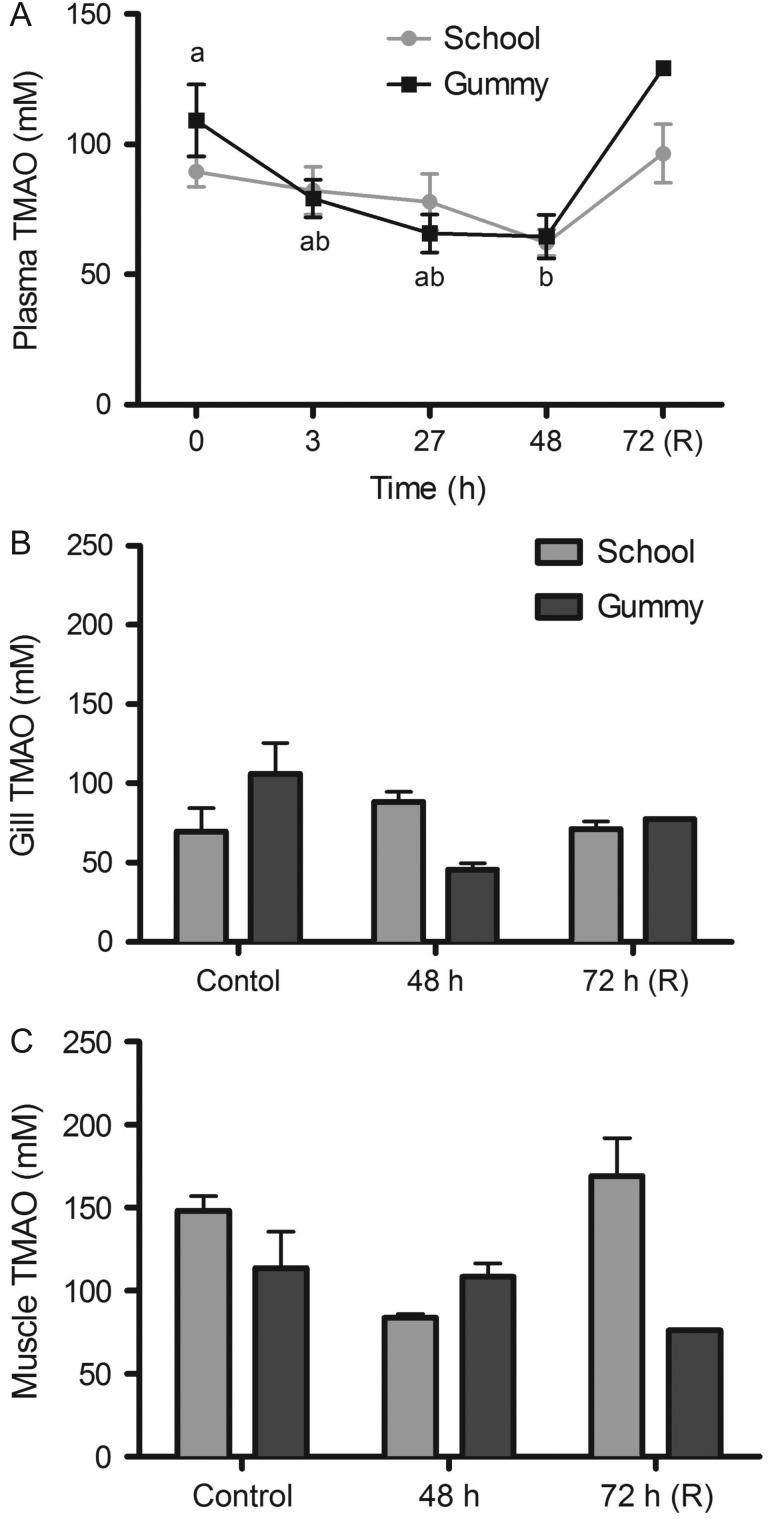
Trimethylamine *N*-oxide (TMAO) concentration (in millimoles per litre; mean values ± SEM) in plasma (**A**), gill (**B**) and white muscle (**C**) in school sharks (*G. galeus*) and gummy sharks (*M. antarcticus*) following exposure to 75% seawater. Different letters indicate statistically significant differences over time (*P* < 0.05; see Materials and methods for species replication numbers). Recovery (R) data are not included in the statistical analyses.

There was an overall decrease in plasma TMAO over time (*P* = 0.0127), and no differences between species (*P* = 0.29). Plasma TMAO concentration decreased significantly (~13%) during the hyposmotic exposure in both species (Fig. 3A; 0 vs. 48 h, *P* = 0.00589). In contrast, there was no change in gill or white muscle TMAO concentration over time in either species, with no significant differences between species, during a hyposmotic exposure (see Fig. 3B, species, *P* = 0.61 and time, *P* = 0.089; and Fig. 3C, species, *P* = 0.54 and time, *P* = 0.19).

### Haematology

In sharks exposed to 75% seawater, there was a significant interaction with species and time for haemoglobin concentrations (Table 2; *P* = 0.015). In school sharks, haemoglobin and haematocrit both declined by ~25% after 3 h in 75% seawater (*P* < 0.001). However, haemoglobin decreased a further 20% after 27 h (*P* < 0.001), whereas haematocrit did not. The haematological response of gummy sharks to 75% seawater was similar to that of school sharks; haemoglobin was 35% lower than the control value after the 3 h (*P* < 0.001), and a further 25% lower after 27 h, whereas haematocrit decreased by 35% after 3 h (*P* < 0.001) but did not change thereafter. The differential response of haemoglobin and haematocrit caused a significant decrease in the mean cell haemoglobin concentration (MCHC) after 27 h in both species (*P* < 0.001).

**Table 2:** Haemoglobin (in grams per litre), haematocrit (as a percentage), mean cell haemoglobin concentration (MCHC; in grams per litre) and whole blood glucose (in millimoles per litre) over time in school sharks (*G. galeus*) and gummy sharks (*M. antarcticus*) exposed to hyposaline conditions

Parameter	Species	Time (h)				
		*t* = 0 (control)	*t* = 3	*t* = 27	*t* = 48	*t* = 72 (recovery)
Haemoglobin*	School	28.1 ± 2.8 (10)^a^	21.1 ± 3.0 (10)^bd^	17.0 ± 2.2 (9)^c^	17.4 ± 2.0 (9)^cd^	33.6 ± 5.6 (4)
	Gummy	31.6 ± 3.2 (8)^a^	20.3 ± 3.2 (8)^b^	15.2 ± 1.9 (7)^c^	15.5 ± 2.4 (5)^c^	16.5 ± 1.6 (2)
Haematocrit	School	16.7 ± 1.4 (10)^a^	12.9 ± 1.4 (10)^b^	11.3 ± 0.8 (9)^b^	11.4 ± 0.9 (9)^b^	22.5 ± 3.5 (4)
	Gummy	19.3 ± 1.8 (8)^a^	12.7 ± 1.6 (8)^b^	13.0 ± 1.2 (7)^b^	12.4 ± 1.7 (5)^b^	15.0 ± 1.0 (2)
MCHC*	School	165 ± 17.4 (10)^a^	150 ± 20.5 (9)^ab^	141 ± 36.9 (9)^b^	148 ± 33.8 (9)^ab^	149 ± 8.9 (4)
	Gummy	163 ± 11.0 (8)^a^	155 ± 25.9 (8)^a^	129 ± 16.0 (7)^b^	125 ± 10.2 (5)^b^	110 ± 4.9 (2)
Whole blood glucose*	School	8.62 ± 0.26 (10)^a^	8.07 ± 0.24 (8)^a^	3.84 ± 0.35 (9)^b^	4.98 ± 0.59 (9)^b^	5.78 ± 0.42 (4)
	Gummy	8.85 ± 0.74 (8)^a^	5.25 ± 0.48 (8)^b^	3.89 ± 0.27 (8)^b^	3.40 ± 0.35 (8)^b^	3.45 ± 0.65 (2)

Data are presented as mean values ± SEM (sample size). Different superscript letters indicate statistically significant differences over time. *Significant species × time interaction.

We observed a significant interaction with species and time in whole blood glucose in sharks exposed to 75% seawater (Table 2; *P* = 0.0025). In gummy sharks, we observed a dramatic decrease in the first 3 h, with blood glucose decreasing by 41% (*P* < 0.001), after which time no further decrease occurred. School sharks experienced a similar decrease in blood glucose (~45%), but this was delayed compared with the gummy sharks and not observed until 27 h into the seawater exposure (*P* < 0.001).

### Heat-shock proteins and ubiquitin

**Figure 4:**
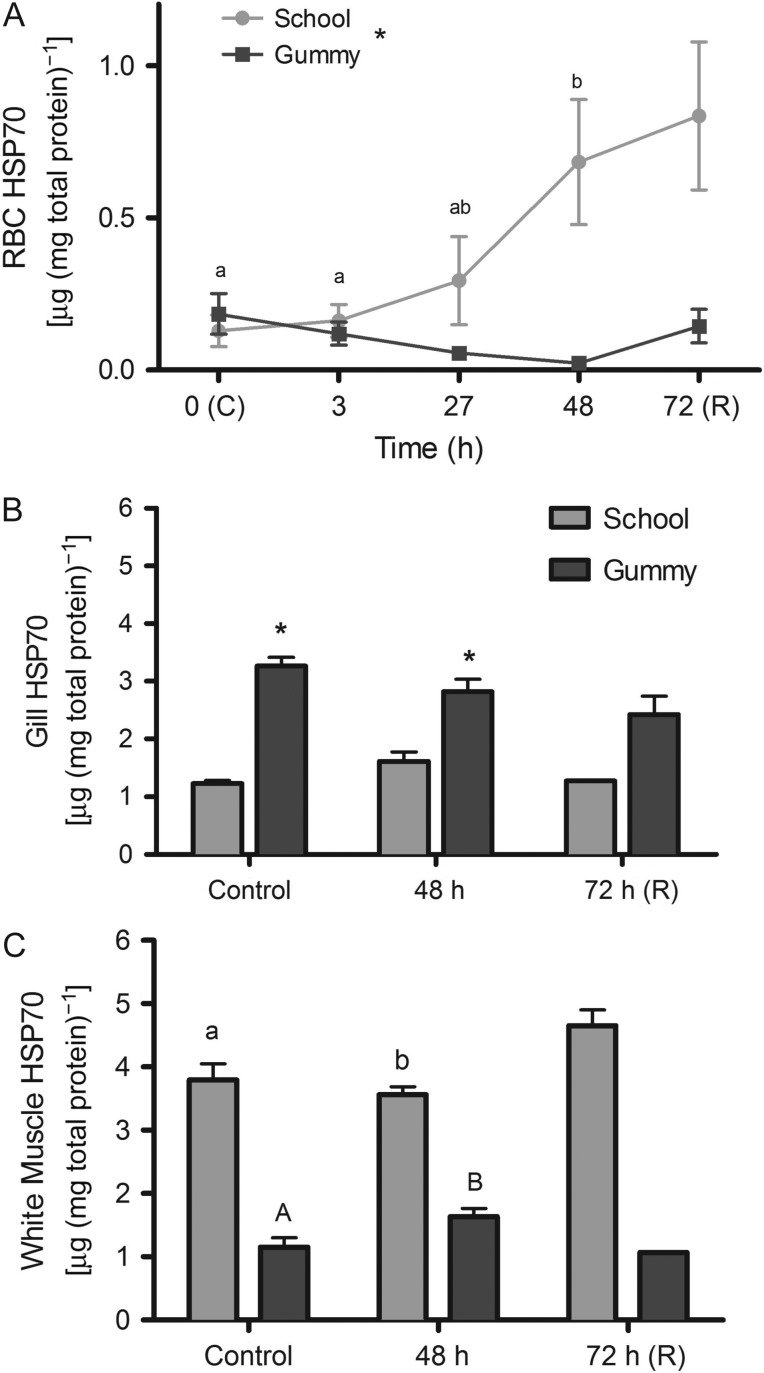
Heat-shock protein 70 (HSP70; in nanagrams of HSP70 per microgram of total protein; mean values ± SEM) in school shark (*G. galeus*) and gummy shark (*M. antarcticus*) red blood cells (RBCs; **A**), gill (**B**) and white muscle (**C**) following exposure to 75% seawater. Different letters indicate statistically significant differences over time. Asterisk indicates significant effect of species (*P* < 0.05; see Materials and methods for species replication numbers). Recovery data are not included in the statistical analyses.

We also measured several HSPs in RBCs, gill and white muscle following hyposmotic exposure, for indications of a cellular stress response. The RBC HSP70 increased by 81% after 48 h of hyposmotic treatment in school sharks only (Fig. 4A; 0 vs. 48 h, *P* < 0.001). In contrast, the RBC HSP70 did not change in gummy sharks over the course of the experiment (Fig. 4A). There was no change in gill HSP70 during hyposmotic conditions in either species (*P* = 0.156), but it is noteworthy that gill HSP70 concentrations were consistently higher in gummy sharks compared with school sharks (Fig. 4B; *P* < 0.0001). We observed significant differences in white muscle HSP70 between species (*P* < 0.0001) and across time (*P* = 0.001). White muscle HSP70 significantly decreased in school sharks after 48 h of hyposmotic exposure (Fig. 4C); however, there was a significant induction of white muscle HSP70 in gummy sharks (Fig. 4C). White muscle HSP70 was significantly higher in school sharks over the course of the experiment.

**Figure 5:**
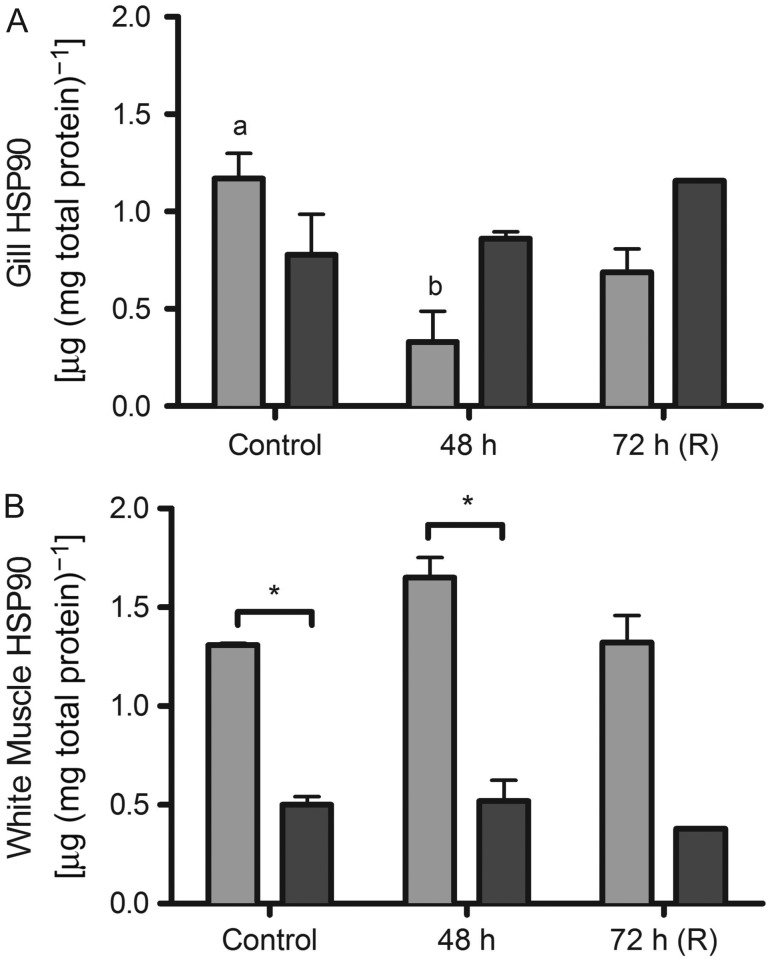
Heat-shock protein 90 (HSP90; in nanograms of HSP90 per microgram of total protein; mean values ± SEM) in school shark (*G. galeus*) and gummy shark (*M. antarcticus*) gill (**A**) and white muscle (**B**) following exposure to 75% seawater. Different letters indicate statistically significant differences over time. Asterisk indicates significant difference between species (*P* < 0.05; see Materials and methods for species replication numbers). Recovery (R) data are not included in the statistical analyses.

Heat shock protein 90 was measured in white muscle and gill tissue and did not change in gummy sharks with hyposmotic exposure (Fig. 5). Similar to HSP70, white muscle HSP90 was significantly higher in school sharks than in gummy sharks (*P* = 0.025; Fig. 5B). There was a significant decrease in school shark HSP90 in the gill (*P* = 0.01; Fig. 5A), but not in the white muscle.

**Figure 6:**
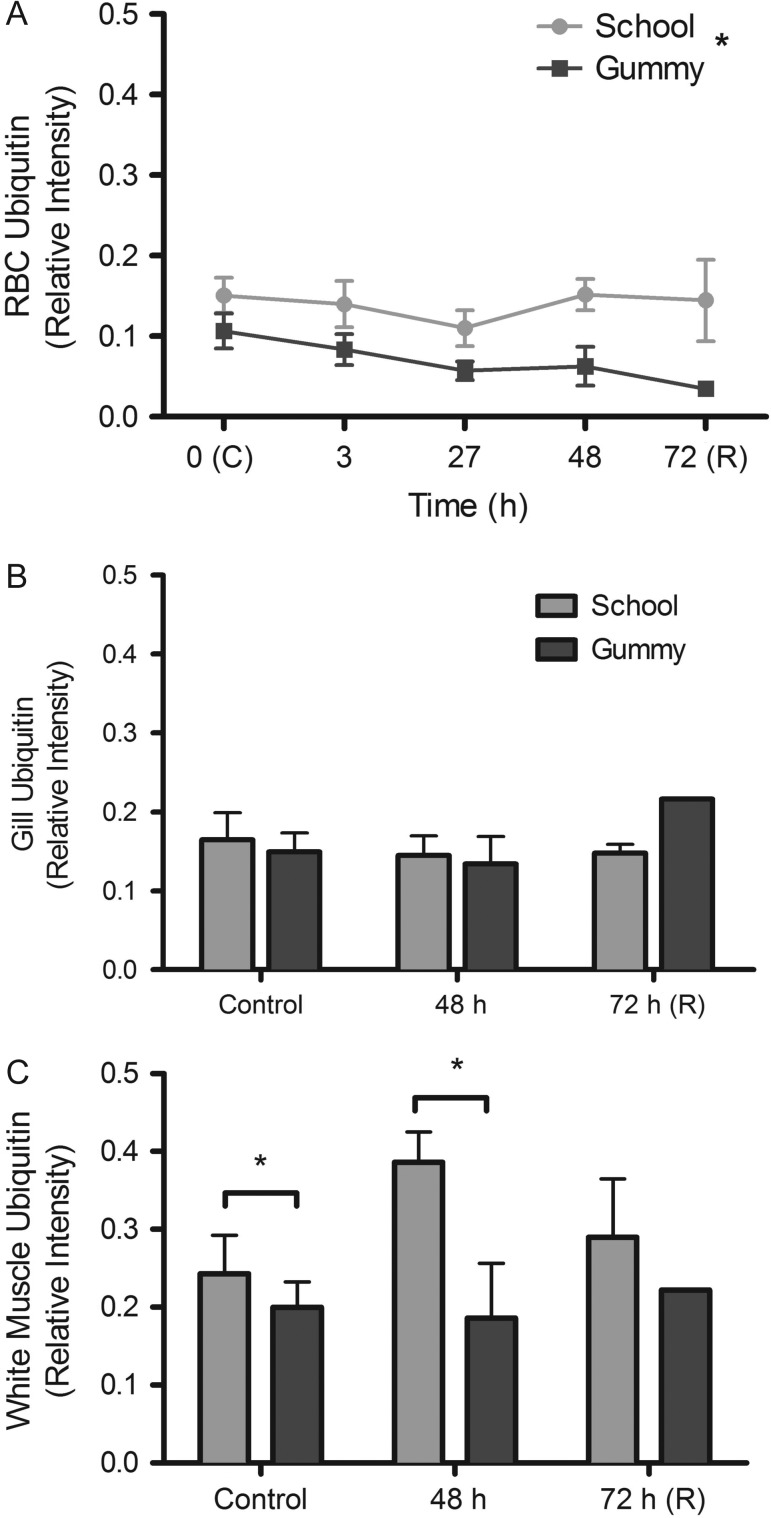
Relative ubiquitin levels (mean values ± SEM) in school shark (*G. galeus*) and gummy shark (*M. antarcticus*) red blood cells (RBCs; **A**), gill (**B**) and white muscle (**C**) following exposure to 75% seawater. Asterisk indicates significant effect of species (*P* < 0.05; see Materials and methods for species replication numbers). Recovery (R) data are not included in the statistical analyses.

Ubiquitin, an indirect indicator of protein damage, did not significantly change with hyposmotic exposure in RBCs (*P* = 0.065), but was maintained at higher levels in school shark RBCs throughout the experiment compared with gummy sharks (*P* = 0.002; Fig. 6A). As was the case for RBCs, neither gill nor white muscle ubiquitin levels changed with hyposmotic exposure in either species (gill, *P* = 0.331; white muscle, *P* = 0.219). However, school sharks had significantly higher levels of ubiquitin in their white muscle compared with gummy sharks (*P* = 0.033; Fig. 6C).

### Oxygen consumption

**Figure 7:**
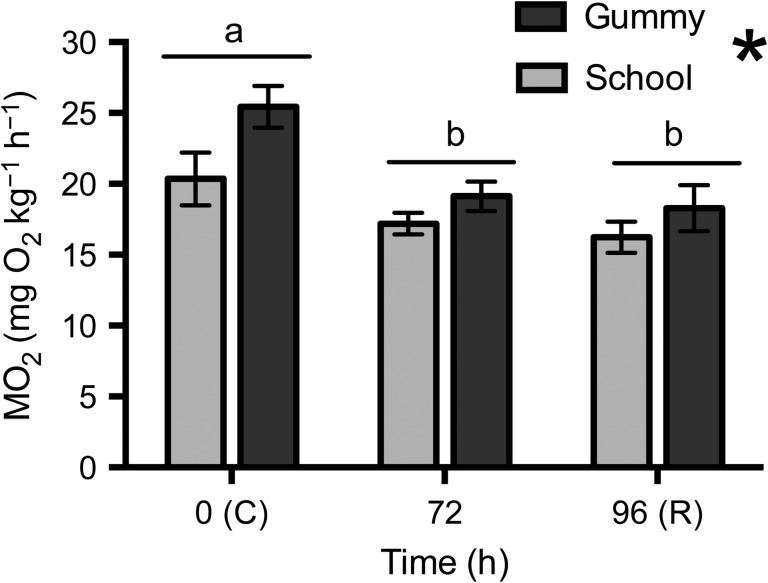
Routine oxygen consumption (MO_2_; in milligrams of O_2_ per kilogram per hour; mean values ± SEM) in school sharks (*G. galeus*) and gummy sharks (*M. antarcticus*) following exposure to 75% seawater. Different letters indicate statistically significant differences over time. Asterisk indicates significant effect of species (*P* < 0.05; school sharks, *n* = 6; gummy sharks, *n* = 5).

Hyposmotic exposure significantly depressed routine oxygen consumption in both school sharks (~15% decrease) and gummy sharks (~25% decrease; *P* = 0.0009; Fig. 7), with no evidence of recovery in either species. There was a significant difference between species (*P* = 0.013), with gummy shark oxygen consumption being higher than that of school sharks, particularly at *t* = 0 (Fig. 7).

## Discussion

The frequency and severity of extreme rainfall events in Tasmania is predicted to increase during the next century (White *et al*., 2013), potentially resulting in more extreme or longer-duration hyposmotic events in coastal estuarine areas. To investigate the effects of hyposalinity on young of the year sharks residing in a nursery, we exposed two species to environmentally relevant hyposmotic (25.8 ppt) conditions for 48 h. We expected to see physiological changes in both species, and our hypothesis was largely supported in that we found that both species showed a similar decrease in aerobic metabolism, MCHC and plasma sodium, glucose and urea concentrations over the 48 h hyposmotic exposure. Interestingly, we found a divergent species response in molecular chaperones after 75% seawater exposure although there was no evidence of protein damage in either species as indicated by stable ubiquitin levels and NKA activity. Our data suggest that both species can physiologically cope with this level of hyposalinity, at least in the short term.

Exposure to acute hyposmotic conditions resulted in the expected significant decrease in plasma osmolality after the first 3 h, and again after 24 h in both species (Table 1). Decreases in elasmobranch plasma osmolality come about primarily through the loss of urea and ions, namely Na^+^ and Cl^−^, as well as the passive influx of water molecules as a result of a salinity decrease (Guffey and Goss, 2014). Dowd *et al*. (2010) found that a 48 h exposure of leopard sharks (*Triakis semifasciata*) to 60% seawater (20.4 ppt) led to a decrease in plasma osmotic concentration, mainly through selective loss of urea and NaCl. We observed a similar pattern in both species; a significant decrease in sodium, chloride and urea over the first 24 h by approximately 20–25%, which could be a selective loss for ion regulation, although we cannot rule out the possibility that water influx may also contribute to this decline. Likewise, the counteracting osmolyte and chemical chaperone, TMAO, also decreased significantly in the plasma of both species after 48 h of hyposmotic exposure.

The elasmobranch gill has an important role in osmoconformation, as it is a major site of ionoregulation (Wright and Wood, 2015). There is much evidence to support a prominent role for the gill in the maintenance of plasma ion concentrations, as the removal of the salt-secreting rectal gland does not cause any changes in Na^+^ and Cl^−^ concentrations (Evans *et al*., 1982; Wilson *et al*., 2002; Wright and Wood, 2015; Deck *et al*., 2016). In contrast, there have been few studies that have investigated the urea concentration in the gills of elasmobranchs. Two studies on dogfish sharks (*S. acanthias*) yielded similar results of ~400 mmol urea (kg wet tissue)^−1^, and there was no effect of hyposmotic exposure on gill urea in *S. acanthias* (Wood *et al*., 2013; MacLellan *et al*., 2015). In the present study, we found slightly lower concentrations of gill urea in both species [school control, 221 ± 35.2 mmol (kg wet tissue)^−1^; gummy control, 302 ± 56.1 mmol (kg wet tissue)^−1^] compared with those reported in dogfish. but a similar lack of effect on urea concentration during the hyposmotic exposure. The lack of decreases in gill urea with lowered salinity may be attributable to a lack of urea permeability in the gills (Pärt *et al*., 1998; Marshall and Grosell, 2006). However, it should be noted that the mechanism for urea retention in elasmobranch gills is not well understood (Wood *et al*., 2013). Gill TMAO also did not change significantly in either species during the hyposaline exposure, indicating that the TMAO concentrations are defended, at least in the gill. Na^+^/K^+^-ATPase activity in the gill remained constant throughout the exposure in both species, suggesting no net increases in Na^+^ uptake across the gills, consistent with other studies in brown-banded bamboo sharks (Cramp *et al*., 2015) and leopard sharks (Dowd *et al*., 2010). Interestingly, gummy shark gill NKA activity was ~50% that of the school sharks, which could potentially limit their ability to cope with more extreme salinity challenges (i.e. <25 ppt), where active Na^+^ uptake may be required.

In contrast to the gill, the white muscle urea concentration of both species decreased significantly during the hyposmotic exposure. The urea concentration dropped by 27% in school sharks and 30% in gummy sharks. These results are consistent with the findings of Steele *et al*. (2005), who found a 30% decrease in the muscle urea concentration of the little skate (*Raja erinacea*) following exposure to dilute seawater, after which the urea concentration returned to control values during a recovery period (Steele *et al*., 2005). Our limited recovery data show a similar trend in muscle urea; thus, it is possible that urea plays a key role in muscle osmoconformation in the gummy and school sharks. As was the case in the gill, the TMAO concentration was defended in the white muscle, and furthermore, the ratio of urea to TMAO was maintained at 2:1 (Yancey, 2005) throughout the exposure in both species.

This maintenance of tissue urea-to-TMAO ratios during hyposaline conditions may prevent cellular/protein damage and remove the signal for HSP induction. We measured HSP (HSP70 and HSP90) levels in RBCs, gill and white muscle and observed significant differences between species in HSP levels with 75% seawater exposure. School sharks induced HSP70 in RBCs but not in the gill or white muscle, although there were high constitutive levels in these tissues over the course of the experiment. Gummy sharks, in contrast, did not induce HSP70 in RBCs or gills, but we observed a modest induction in white muscle. Taken together, hyposmotic exposure resulted in a tissue- and species-specific cellular stress response and, at least for gill and white muscle, where we have urea-to-TMAO ratios, HSP induction appears to be independent of TMAO and urea concentrations (see next paragraph). Dogfish (*S. acanthias*) exposed to similar low-saline conditions also induced HSP70 but in the gills only, with a concomitant increase in ubiquitin levels (MacLellan *et al*., 2015), the latter being an indirect indication of protein damage. The conclusion from the dogfish study was that hyposmotic exposure caused protein damage at the gill, resulting in HSP70 induction, thus preserving gill function. In the school and gummy sharks of our study, in contrast, ubiquitin levels remained constant throughout the exposure in both species, regardless of HSP induction. The lack of correlation with ubiquitin levels and HSPs could mean: (i) no damage has occurred with hyposmotic exposure; (ii) the ubiquitin assay, which is also reflective of normal protein turnover (Houlihan *et al*., 1995), is not sensitive enough to detect damage; or (iii) both constitutive and inducible HSP levels are adequate to prevent any damage. Regardless, we did observe species differences in the HSP70 response, and overall, the school sharks appear to mount a greater cellular stress response than the gummy sharks. A cellular stress response, at least in RBCs, may be an adaptive mechanism for life in the estuary, especially given that these species differences in HSPs were not reflected in our physiological measures. Such a stress response is reminiscent of intertidal snails (genus *Tegula*), with those living high on the intertidal zone and subject to more environmental variation having a greater magnitude heat shock response than species living lower on the intertidal zone (Tomanek and Somero, 1999).

TMAO serves as a protective chemical chaperone (Welch and Brown, 1996), in addition to its role as an osmolyte, and there is evidence in elasmobranchs that it has a reciprocal role with molecular chaperones, such as HSPs, to maintain cellular protein stability/function during times of stress (Villalobos and Renfro, 2007; Kolhatkar *et al*., 2014; MacLellan *et al*., 2015). If TMAO is lost as the animal attempts to osmoconform to a new lower salinity, HSPs may be more likely to be induced to take over the chaperone function. However, we found no evidence of chaperone reciprocity in these two species during a hyposmotic exposure. Nonetheless, it is important to note that TMAO concentrations were largely maintained (exception in plasma) with our hyposmotic exposure in both species. TMAO concentration, together with constitutive and induced HSPs and lack of obvious damage, suggest that both species are able to cope with the present level of hyposmotic stress, and potentially, able to cope with longer or more intense hyposmotic events.

Hyposmotic exposure had a significant effect on oxygen transport and aerobic metabolism in both species. After 3 h in 75% seawater, we observed significant changes in the blood in both sharks. Haematocrit and haemoglobin both decreased by ~40% after 48 h in 75% seawater, a common response in marine elasmobranchs faced with lower salinity (Goldstein and Forster, 1971; Chan and Wong, 1977; Cooper and Morris, 2004; Cramp *et al*., 2015). However, the slight differences in the absolute decrease of haemoglobin compared with haematocrit resulted in a significant decrease in MCHC in both species. A decrease in MCHC would decrease the ability to transport oxygen and potentially affect aerobic metabolism. Indeed, we found a significant decline in routine oxygen consumption in both species (gummy sharks, −25%; school sharks, −15%) after 24 h in 75% seawater. Guffey and Goss (2014) also found a decrease in oxygen consumption after 24 h of hyposmotic conditions in *S. acanthias*. A loss of plasma osmolytes (e.g. NaCl, urea and TMAO) can also alter the oxygen-carrying capacity and binding affinity of haemoglobin, further reducing oxygen transport and consumption. A loss of urea can alter ATP binding to haemoglobin, thereby reducing the haemoglobin–O_2_ affinity in dogfish (Weber, 1983; Weber *et al*., 1983), and a similar effect of low urea on haemoglobin–O_2_ affinity was also observed in Port Jackson sharks at 75 and 50% seawater (Cooper and Morris, 2004). Thus, it is possible that low plasma osmolality/urea may lower haemoglobin–O_2_, ultimately lowering the rate of oxygen consumption in school and gummy sharks. It is also possible that these species are reducing gill perfusion to restrict ion loss, which in turn decreases oxygen uptake; the so-called osmorespiratory compromise (Randall *et al*., 1972; Gonzalez and McDonald, 1992; Sardella and Brauner, 2007). This is well documented in teleost fish, but has yet to be tested in elasmobranchs. We also observed a significant decline in whole blood glucose with hyposaline challenge after 3 h in gummy sharks and after 24 h in school sharks. It is not clear from the data whether glucose is decreasing from a plasma dilution or whether it is being taken up by the tissues as a substrate for energy production via glycolysis in the face of decreased aerobic metabolism.

After 24 h recovery in 100% seawater, oxygen consumption did not return to control values in either species. Interestingly, school shark haemoglobin and haematocrit values from our limited recovery samples appear to be higher than the control values, but this had no effect on oxygen consumption. Future research should investigate recovery times in these species, as a loss of metabolic capacity would restrict growth, movement, reproduction, etc. If the frequency/duration of hyposmotic events increases as predicted, and recovery times are long, this may restrict the use of the estuarine environment.

The variation in physiological responses we observed between the school and gummy sharks may help to predict their distribution and ability to use estuaries with fluctuating salinity (Heupel *et al*., 2003; Heupel and Simpfendorfer, 2008; Udyawer *et al*., 2013; Yates *et al*., 2015). It is possible that school sharks are hardwired to respond to environmental variation with a robust cellular stress response. Stress-related short-term changes in gene expression (e.g. induction of *hsp* genes) are correlated with the long-term modification of gene expression (López-Maury *et al*., 2008), suggesting that HSP70 induction and higher constitutive levels of HSPs in the white muscle of school sharks may be an evolutionary adaptation that allows them to cope with the environmental variability they encounter in estuaries (Tomanek and Somero, 1999, 2000). The higher gill NKA activity relative to gummy sharks may also better equip school sharks to respond to salinity change; a promising possibility for a species that is listed as ‘conservation dependent’. In contrast, the higher magnitude of HSP70 induction in school sharks could be indicative of a more severe stress response or a protective mechanism; however, there do not appear to be any major physiological ramifications to this enhanced cellular stress response.

Understanding physiological limits to fluctuating salinity and the underlying mechanisms, particularly in juvenile sharks, will be important for their conservation. Our data suggest that if extreme rainfall events become more frequent or occur over a longer duration, these species may be restricted from using the estuarine environment, putting them at greater risk for predation. There is already emerging evidence that gummy sharks are not found as frequently as school sharks in the Pittwater Estuary, particularly when there are heavy rainfall events. Specifically, acoustic monitoring of electronically tagged school and gummy sharks indicates that school sharks primarily stay in the Pittwater Estuary during the wet summer months, whereas gummy sharks more frequently move out into the adjoining Frederick Henry Bay (Jaime D. McAllister, Adam Barnett, Kátya Abrantes and Jayson M. Semmens, unpublished observations) where salinity is more stable (mean 33.9 ± 0.2‰ from 1991 to 1994; Crawford and Mitchell, 1999). Natural changes in the salinity of shark nurseries are intrinsically linked with changes in temperature, and it would be worth examining the stress response and coping mechanisms of these shark species to a combination of osmotic and thermal stress (Morrissey and Gruber, 1993; Grubbs *et al*., 2005; Heupel *et al*., 2007; Knip *et al*., 2010).

## References

[cow036C1] Awruch CA, Simpfendorfer C, Pankhurst NW (2011) Evaluation and use of a portable field kit for measuring whole-blood lactate in sharks. Mar Freshw Res 62: 694–699.

[cow036C2] Branstetter S (1990) Early life-history implications of selected carcharhinoid and lamnoid sharks of the northwest Atlantic In Pratt HL, Gruber SH, Taniuchi T, eds, Elasmobranchs as Living Resources: Advances in the Biology, Ecology, Systematics, and the Status of the Fisheries, NOAA Technical Report 90, National Marine Fisheries Service, Silver Spring, MD. pp 17–28.

[cow036C3] Castro JI (1993) The shark nursery of Bulls Bay, South Carolina, with a review of the shark nurseries of the southeastern coast of the United States In Demski LS, Wourms JP, eds, The Reproduction and Development of Sharks, Skates, Rays and Ratfishes. Springer, Dordrecht, pp 37–48.

[cow036C4] Chan DKO, Wong TM (1977) Physiological adjustments to dilution of the external medium in the lip-shark, *Hemiscyllium plagiosum* (Bennett). III. Oxygen consumption and metabolic rates. J Exp Zool 200: 97–102.

[cow036C5] Clark TD, Eliason EJ, Sandblom E, Hinch SG, Farrell AP (2008) Calibration of a hand-held haemoglobin analyser for use on fish blood. J Fish Biol 73: 2587–2595.

[cow036C6] Cooper AR, Morris S (2004) Haemoglobin function and respiratory status of the Port Jackson shark, *Heterodontus portusjacksoni*, in response to lowered salinity. J Comp Physiol B 174: 223–236. 14712328 10.1007/s00360-003-0405-1

[cow036C7] Cramp RL, Hansen MJ, Franklin CE (2015) Osmoregulation by juvenile brown-banded bamboo sharks, *Chiloscyllium punctatum*, in hypo- and hyper-saline waters. Comp Biochem Physiol A Mol Integr Physiol 185: 107–114. 25868436 10.1016/j.cbpa.2015.04.001

[cow036C8] Crawford C, Mitchell I (1999) Physical and chemical parameters of several oyster growing areas in Tasmania, Technical Report Series, Number 4, Tasmanian Aquaculture and Fisheries Institute, University of Tasmania, Hobart. pp 101.

[cow036C9] Deck CA, Bockus AB, Seibel BA, Walsh PJ (2016) Effects of short-term hyper- and hypo-osmotic exposure on the osmoregulatory strategy of unfed North Pacific spiny dogfish (*Squalus suckleyi*). Comp Biochem Physiol A Mol Integr Physiol 193: 29–35. 26686463 10.1016/j.cbpa.2015.12.004

[cow036C10] Dowd WW, Renshaw GMC, Cech JJ, Kültz D (2010) Compensatory proteome adjustments imply tissue-specific structural and metabolic reorganization following episodic hypoxia or anoxia in the epaulette shark (*Hemiscyllium ocellatum*). Physiol Genomics 42: 93–114. 20371547 10.1152/physiolgenomics.00176.2009 PMC2888556

[cow036C11] Evans DH, Oikari A, Kormanik GA, Mansberger L (1982) Osmoregulation by the prenatal spiny dogfish, *Squalus acanthias*. J Exp Biol 101: 295–305.

[cow036C52] Fowler SL, Hamilton D, Currie S (2009) A comparison of the heat shock response in juvenile and adult rainbow trout (*Oncorhynchus mykiss*) - implications for increased thermal sensitivity with age. Can J Fish Aquat Sci 66: 91–100.

[cow036C12] Forster RP, Goldstein L (1976) Intracellular osmoregulatory role of amino acids and urea in marine elasmobranchs. Am J Physiol 230: 925–931. 1267026 10.1152/ajplegacy.1976.230.4.925

[cow036C13] French RP, Lyle J, Tracey S, Currie S, Semmens JM (2015) High survivorship after catch-and-release fishing suggests physiological resilience in the endothermic shortfin mako shark (*Isurus oxyrinchus*). Conserv Physiol 3: cov044; 10.1093/conphys/cov044. 27303650 10.1093/conphys/cov044 PMC4778490

[cow036C14] Georgopoulos C, Welch WJ (1993) Role of the major heat shock proteins as molecular chaperones. Annu Rev Cell Biol 9: 601–634. 8280473 10.1146/annurev.cb.09.110193.003125

[cow036C15] Goldstein L, Forster RP (1971) Urea biosynthesis and excretion in freshwater and marine elasmobranchs. Comp Biochem Physiol B 39: 415–421. 5119910 10.1016/0305-0491(71)90186-6

[cow036C16] Gonzalez RJ, McDonald DG (1992) The relationship between oxygen consumption and ion loss in a freshwater fish. J Exp Biol 163: 317–332. 10.1242/jeb.190.1.95 9317409

[cow036C17] Grubbs RD, Musick JA, Conrath CL, Romine JG (2005) Long-term movements, migration and temporal delineation of a summer nursery for juvenile sandbar sharks in the Chesapeake Bay region. *American Fisheries Society Symposium Series* 50: 87–107.

[cow036C18] Guffey SC, Goss GG (2014) Time course of the acute response of the North Pacific spiny dogfish shark (*Squalus suckleyi*) to low salinity. Comp Biochem Physiol A Mol Integr Physiol 171: 9–15. 24518388 10.1016/j.cbpa.2014.02.004

[cow036C19] Heupel MR, Simpfendorfer C (2008) Movement and distribution of young bull sharks *Carcharhinus leucas* in a variable estuarine environment. Aquat Biol 1: 277–289.

[cow036C20] Heupel MR, Simpfendorfer CA, Hueter RE (2003) Running before the storm: blacktip sharks respond to falling barometric pressure associated with Tropical Storm Gabrielle. J Fish Biol 63: 1357–1363.

[cow036C21] Heupel MR, Carlson JK, Simpfendorfer CA (2007) Shark nursery areas: concepts, definition, characterization and assumptions. Mar Ecol Prog Ser 337: 287–297.

[cow036C22] Houlihan DF, Carter CG, McCarthy ID (1995) Protein turnover in animals In Walsh PJ, Wright PA, eds, Nitrogen Metabolism and Excretion. CRC Press, Boca Raton, pp 1–32.

[cow036C53] IPCC, 2013: Climate Change 2013: The Physical Science Basis Contribution of Working Group I to the fifth Assessment Report of the Intergovernmental Panel on Climate Change [Stocker T.F., Qin D., Plattner G.-K., Tignor M., Allen S.K., Boschung J., Nauels A., Xia Y., Bex V. and Midgley P.M.(eds.)]. Cambridge University Press, Cambridge, United Kingdom and New York, NY, USA, 1535pp.

[cow036C23] Knip DM, Heupel MR, Simpfendorfer CA (2010) Sharks in nearshore environments: models, importance, and consequences. Mar Ecol Prog Ser 402: 1–11.

[cow036C24] Kolhatkar A, Robertson CE, Thistle ME, Gamperl AK, Currie S (2014) Coordination of chemical (trimethylamine oxide) and molecular (heat shock protein 70) chaperone responses to heat stress in elasmobranch red blood cells. Physiol Biochem Zool 87: 652–662. 25244377 10.1086/676831

[cow036C25] Kumar R (2009) Role of naturally occurring osmolytes in protein folding and stability. Arch Biochem Biophys 491: 1–6. 19769937 10.1016/j.abb.2009.09.007

[cow036C26] López-Maury L, Marguerat S, Bähler J (2008) Tuning gene expression to changing environments: from rapid responses to evolutionary adaptation. Nat Rev Genet 9: 583–593. 18591982 10.1038/nrg2398

[cow036C27] MacLellan RJ, Tunnah L, Barnett D, Wright PA, MacCormack T, Currie S (2015) Chaperone roles for TMAO and HSP70 during hyposmotic stress in the spiny dogfish shark (*Squalus acanthias*). J Comp Physiol B 185: 729–740. 26050212 10.1007/s00360-015-0916-6

[cow036C28] Marshall WS, Grosell M (2006) Ion transport, osmoregulation and acid base balance In Evans DH, Claiborne JB, eds, The Physiology of Fishes. CRC Press, Boca Raton, pp 177–230.

[cow036C54] McCormick SD (1993) Methods for nonlethal gill biopsy and measurement of Na^+^,K^+^-ATPase activity. Can J Fish Aquat Sci 50: 656–658.

[cow036C29] Morrissey JF, Gruber SH (1993) Habitat selection by juvenile lemon sharks, *Negaprion brevirostris*. Environ Biol Fish 38: 311–319.

[cow036C30] Pärt P, Wright PA, Wood CM (1998) Urea and water permeability in dogfish (*Squalus acanthias*) gills. Comp Biochem Physiol A Mol Integr Physiol 119: 117–123. 11253775 10.1016/s1095-6433(97)00400-5

[cow036C55] Rahmatullah H, Boyde TRC (1980) Improvements in the determination of urea using diacetyl monoxide; methods with and without deproteinisation. Clin Chim Acta 107: 3–9. 7428175 10.1016/0009-8981(80)90407-6

[cow036C31] Randall DJ, Baumgarten D, Malyusz M (1972) The relationship between gas and ion transfer across the gills of fishes. Comp Biochem Physiol A 41: 629–637. 4401733 10.1016/0300-9629(72)90017-5

[cow036C32] Sardella BA, Brauner CJ (2007) The osmo-respiratory compromise in fish; the effects of physiological state and the environment In Fernandes MN, Rantin FT, Glass ML, Kapoor BG, eds, Fish Respiration and Environment. Science Publisher, Inc., Enfield, NH, USA, pp 147–165.

[cow036C33] Schlaff AM, Heupel MR, Simpfendorfer CA (2014) Influence of environmental factors on shark and ray movement, behaviour and habitat use: a review. Rev Fish Biol Fish 24: 1089–1103.

[cow036C34] Schmidt-Nielsen B, Truniger B, Rabinowitz L (1972) Sodium-linked urea transport by the renal tubule of the spiny dogfish *Squalus acanthias*. Comp Biochem Physiol A 42: 13–25. 4402703 10.1016/0300-9629(72)90360-x

[cow036C35] Smith HW (1931) The absorption and excretion of water and salts by the elasmobranch fishes II. Marine elasmobranchs. Am J Physiol 98: 296–310.

[cow036C36] Steele SL, Yancey PH, Wright PA (2005) The little skate (*Raja erinacea*) exhibits an extrahepatic ornithine urea cycle in the muscle and modulates nitrogen metabolism during low-salinity challenge. Physiol Biochem Zool 78: 216–226. 15778941 10.1086/427052

[cow036C37] Stevens JD, West GJ (1997) Investigation of school and gummy shark nursery areas in south eastern Australia, Fisheries Research and Development Corporation, Australia, Final Report: Project 93/061, pp 1–76.

[cow036C38] Tomanek L, Somero GN (1999) Evolutionary and acclimation-induced variation in the heat-shock responses of congeneric marine snails (genus *Tegula*) from different thermal habitats: implications for limits of thermotolerance and biogeography. J Exp Biol 202: 2925–2936. 10518474 10.1242/jeb.202.21.2925

[cow036C39] Tomanek L, Somero GN (2000) Time course and magnitude of synthesis of heat-shock proteins in congeneric marine snails (genus *Tegula*) from different tidal heights. Physiol Biochem Zool 73: 249–256. 10801403 10.1086/316740

[cow036C40] Tunnah L, MacKellar SRC, Barnett DA, MacCormack TJ, Stehfest KM, Morash AJ, Semmens JM, Currie S (2016) Physiological responses to hypersalinity correspond to nursery ground usage in two inshore shark species (*Mustelus antarcticus* and *Galeorhinus galeus*). J Exp Biol 219: 2028–2038. 27207636 10.1242/jeb.139964

[cow036C41] Udyawer V, Chin A, Knip DM, Simpfendorfer CA, Heupel MR (2013) Variable response of coastal sharks to severe tropical storms: environmental cues and changes in space use. Mar Ecol Prog Ser 480: 171–183.

[cow036C42] Villalobos AR, Renfro JL (2007) Trimethylamine oxide suppresses stress-induced alteration of organic anion transport in choroid plexus. J Exp Biol 210: 541–552. 17234624 10.1242/jeb.02681

[cow036C43] Weber RE (1983) TMAO (trimethylamine oxide)-independence of oxygen affinity and its urea and ATP sensitivities in an elasmobranch hemoglobin. J Exp Zool 228: 551–554. 6663264 10.1002/jez.1402280315

[cow036C44] Weber RE, Wells RM, Rossetti JE (1983) Allosteric interactions governing oxygen equilibria in the haemoglobin system of the spiny dogfish, *Squalus acanthias*. J Exp Biol 103: 109–120. 6854197 10.1242/jeb.103.1.109

[cow036C45] Welch WJ, Brown CR (1996) Influence of molecular and chemical chaperones on protein folding. Cell Stress Chaperones 1: 109–115. 9222596 10.1379/1466-1268(1996)001<0109:iomacc>2.3.co;2 PMC248462

[cow036C46] White CJ, McInnes KL, Cechet RP, Corney SP, Grose MR, Holz GK, Katzfey JJ, Bindoff NL (2013) On regional dynamical downscaling for the assessment and projection of future temperature and precipitation extremes across Tasmania, Australia. Clim Dyn 41: 1–21.

[cow036C47] Wilson JM, Morgan JD, Vogl AW, Randall DJ (2002) Branchial mitochondria-rich cells in the dogfish *Squalus acanthias*. Comp Biochem Physiol A Mol Integr Physiol 132: 365–374. 12020652 10.1016/s1095-6433(02)00042-9

[cow036C48] Wood CM, Liew HJ, De Boeck G, Walsh PJ (2013) A perfusion study of the handling of urea and urea analogues by the gills of the dogfish shark (*Squalus acanthias*). PeerJ 1: e33. 23638369 10.7717/peerj.33 PMC3628372

[cow036C49] Wright PA, Wood CM (2015) Regulations of ions, acid-base and nitrogenous wastes in elasmobranchs In: Shadwick RE, Farrell AP, Brauner CJ, eds, Fish Physiology vol 34B: Physiology of Elasmobranch Fishes: Internal Processes. Academic Press, New York, pp 279–345.

[cow036C50] Yancey PH (2005) Organic osmolytes as compatible, metabolic and counteracting cytoprotectants in high osmolarity and other stresses. J Exp Biol 208: 2819–2830. 16043587 10.1242/jeb.01730

[cow036C51] Yates PM, Heupel MR, Tobin AJ, Simpfendorfer CA (2015) Ecological drivers of shark distributions along a tropical coastline. PLoS One 10: e0121346. 25853657 10.1371/journal.pone.0121346 PMC4390147

